# A Cross-Sectional Analysis of Deprescribing Curricular Exposure, Attitudes, and Preparedness Among Undergraduate Pharmacy Students in Saudi Arabia

**DOI:** 10.3390/pharmacy14050124

**Published:** 2026-08-21

**Authors:** Lina Altayeb, Hend Talkhan, Rayah Asiri, Rawan Aloqran, Arwa Al Saeed, Khalid Orayj

**Affiliations:** 1 Department of Clinical Pharmacy, College of Pharmacy, King Khalid University, Abha 61421, Saudi Arabia; 2 College of Pharmacy, King Khalid University, Abha 61421, Saudi Arabia

**Keywords:** pharmacy, undergraduate students, deprescribing, attitude, perception, preparedness, confidence, Saudi Arabia

## Abstract

Objectives: This study aimed to assess pharmacy students’ curricular exposure to deprescribing, their attitudes and preparedness to perform core deprescribing steps, and their understanding of interprofessional roles in the deprescribing process. Methods: A cross-sectional online survey that included undergraduate pharmacy students in their fifth or sixth year of study, who were enrolled in Saudi universities during the academic year 2025/2026. A 43-item self-administered validated questionnaire was used. The independent-samples *t*-test was used to examine differences in attitudes and preparedness among participants via SPSS 27. Results: In total, 367 students from 23 universities participated in the study; 54.0% were female, and 62.1% were in their fifth year. Familiarity with the term ‘deprescribing’ was reported by 55.3% of participants. Participants demonstrated highly positive attitudes toward the benefits of deprescribing, moderate toward misconceptions, and low toward barriers, with mean percentage scores of 85.08%, 66.50%, and 41.93%, respectively. Participants indicated moderately positive perceptions toward the non-experiential curriculum preparedness to identify and practice deprescribing and self-perception of the ability to perform core deprescribing steps, with mean percentage scores of 70.2% and 77.24%, respectively. Statistically significant differences were observed in the perception of curriculum preparedness by gender (*p* = 0.038), familiarity with the term deprescribing (*p* < 0.001), and exposure to deprescribing in the required pharmacy curriculum (*p* < 0.001). Participants perceived clinical pharmacists as having the greatest role across most stages of the deprescribing process. Conclusions: Undergraduate pharmacy students in Saudi Arabia recognize the value of deprescribing but have limited curricular exposure and moderate perceived preparedness to deprescribe.

## 1. Introduction

Polypharmacy and potentially inappropriate medication (PIM) use have become major public health concerns worldwide, particularly among older adults and patients with multiple chronic conditions [1]. Polypharmacy is associated with increased risks of adverse drug reactions, drug-drug interactions, medication non-adherence, falls, cognitive impairment, hospitalization, and increased healthcare costs [2]. These risks are further aggravated by the growing prevalence of multimorbidity and the reliance on disease-specific clinical guidelines that may not adequately address the complexities of patients receiving multiple medications [3].

To address the negative impact of polypharmacy and PIM use, deprescribing has emerged as an important patient-centered clinical strategy [4,5]. The term deprescribing refers to the systematic and supervised process of tapering or discontinuing medications when their potential harm outweighs their current or future benefits [6,7]. Previous studies have demonstrated that deprescribing interventions, when appropriately implemented, can safely reduce medication burden, minimize medication-related harm, and improve clinical outcomes in selected patient populations [8,9].

As healthcare systems strive to integrate deprescribing into routine clinical practice, healthcare education programs are expected to equip future professionals with the competencies necessary to participate effectively in medication optimization [10]. Within multidisciplinary healthcare teams, pharmacists are particularly well positioned to contribute to deprescribing initiatives because of their extensive training in pharmacotherapy, medication review, drug interactions, and patient counseling [11]. Consequently, it is important to examine whether undergraduate pharmacy education adequately prepares future pharmacists to identify PIMs, evaluate risk-benefit profiles, communicate deprescribing recommendations, and participate in shared decision-making with patients and healthcare teams [10,12].

International educational literature, although limited in volume, has shown that pharmacy students generally demonstrate positive attitudes toward deprescribing and recognize its importance in clinical practice [13,14]. However, studies have also reported considerable variability in students’ confidence, practical preparedness, and self-efficacy regarding deprescribing activities. Many students feel insufficiently prepared to recommend discontinuing medication, communicate deprescribing plans to physicians, or address patient concerns related to stopping medications [12,15]. Several factors, such as year of study, extent of curricular exposure, clinical training experiences, and previous formal education related to deprescribing, may influence pharmacy students’ attitudes, confidence, and willingness to engage in deprescribing practices [10,15]. Understanding these factors may help educators identify curricular deficiencies and improve educational strategies aimed at strengthening deprescribing competencies among future pharmacists [10].

In Saudi Arabia, polypharmacy and PIM use are common among older adults across different settings [16,17]; however, the implementation of deprescribing within clinical practice is still limited [18]. Several barriers, including limited awareness of deprescribing guidelines, concerns regarding treatment discontinuation, and challenges in interprofessional communication, have been reported [18]. Therefore, preparing future pharmacists with adequate knowledge, skills, and communication competencies in deprescribing represents an important step toward improving medication optimization practices within the Saudi healthcare system [19].

Currently, there are no explicit, mandatory national curricular requirements for deprescribing. However, the proposed Saudi pharmacy competency framework [20] and pharmacotherapy didactic curriculum toolkit [21] incorporate foundational skills and topics relevant to deprescribing, such as safe medication use in older people, patient assessment, medication reconciliation and review, and communication with patients and healthcare professionals. Similarly, the Saudi Pharmacist Licensure Examination blueprint [22] includes several competencies that underpin deprescribing practice, such as obtaining a comprehensive medication history, identifying and prioritizing drug-related problems, counseling patients, and developing safe and effective medication plans for special populations, including geriatrics. However, the blueprint does not specifically use the term “deprescribing”.

Little is known regarding Saudi pharmacy students’ experience with deprescribing education, their confidence, and perceptions of deprescribing. A single study surveyed 253 PharmD students across all years of study and reported moderate confidence, high positive perception, and a relatively high familiarity with the deprescribing concept [23]. Interestingly, only 54.7% of participants indicated that they were willing to enroll in a workshop on deprescribing. A more extensive understanding of students’ perspectives is crucial for determining how educational experiences shape students’ views and readiness for clinical practice. Therefore, this study sought to assess pharmacy students’ curricular exposure, attitudes, and preparedness to perform deprescribing activities, and their understanding of interprofessional roles in deprescribing. In addition, the study aimed to explore factors associated with students’ attitudes and preparedness.

## 2. Materials and Methods

### 2.1. Study Setting

This was a cross-sectional online survey that included undergraduate pharmacy students who were enrolled in Saudi universities during the academic year 2025/2026. Students in their fifth or sixth year of study were eligible for inclusion to ensure exposure to most clinical, didactic, and experiential courses.

### 2.2. Sampling Method

Potential participants were selected using the convenience sampling method. Cochran’s formula for an unknown population was used to calculate the sample size, given a 0.05 margin of error, 95% confidence interval, and an estimated proportion of 50%. The recommended sample size was 385.

### 2.3. Data Collection

Data collection took place between January and April 2026. A self-administered validated questionnaire was adapted from previous studies [24,25] (Supplementary Materials). Three professionals with expertise in clinical pharmacy practice and education evaluated the questionnaire’s content validity. The questionnaire was then piloted in a sample of fifteen students to assess its clarity, relevance, and appropriateness. Minor refinements were made in response to the pilot, such as reordering the scale options. The questionnaire was designed using Google Forms and disseminated to the Telegram groups of Saudi pharmacy students. These Telegram groups were created by students affiliated with their respective colleges to facilitate academic communication, peer support, and dissemination of student-related information (e.g., extracurricular activities, research opportunities, and educational resources.

Forty-three items in total, divided into three parts, made up the questionnaire:

- Part one: seven closed-ended questions about the participant’s demographic and educational background, including age, gender, study level, plans for future employment, and settings in which the Introductory Pharmacy Practice Experience (IPPE) and/or Advanced Pharmacy Practice Experience (APPE) took place (e.g., community pharmacy, hospital, clinic-based pharmacy, eldercare, long-term care). Eldercare is an umbrella term that encompasses all health, social, and psychological services provided to older adults in a variety of settings, including care homes and the older adult’s own home. Long-term care refers to the wide range of services provided over extended periods to individuals with, or at risk of, functional decline or disability.
- Part two: four closed-ended questions about familiarity with the term ‘deprescribing’, required and elective curricular exposure to deprescribing principles, and the teaching activities during which instruction took place. A commonly accepted definition of deprescribing was provided after the first question to inform participants and enhance the accuracy of answers.
- Part three: thirty-two closed-ended questions about (A) the participant’s attitude toward deprescribing, including its benefits, barriers and common misconceptions (sixteen items, 5-point Likert scale ranging from strongly agree to strongly disagree), (B) perception of non-experiential curriculum role in preparing participants to identify and deprescribe PIMs (four items, 5-point Likert scale ranging from strongly agree to strongly disagree), (C) self-perceived preparedness to implement the seven core steps of deprescribing [24] (seven items, 5-point Likert scale ranging from most prepared to least prepared), and (D) the roles played by professional groups (i.e., physicians, nurses, and pharmacists) in the deprescribing process (five items).

### 2.4. Data Analysis

Data were imported into Microsoft Excel, then analyzed using SPSS version 27. Descriptive statistics were used to summarize the data, including frequencies and proportions for categorical variables.

Likert scale responses were scored from 1 (strongly disagree) to 5 (strongly agree). Reverse coding was applied to negatively worded items, where 1 corresponded to strongly agree and 5 to strongly disagree. Responses were ranked from 1 (least prepared) to 5 (most prepared) on the self-perceived preparedness to deprescribe scale.

The distribution of scale scores was assessed for normality using the Shapiro–Wilk test, skewness, kurtosis, and visual inspection of scatter plots. For each scale, the mean scale score ± standard deviation and the mean percentage score were calculated. The mean percentage score was calculated using the formula: (student’s total score ÷ maximum possible scale score) × 100. The mean percentage score was then interpreted according to Bloom’s cut-off criteria as follows: low (<60%), moderate (60–80%), and high (>80%).

An independent samples *t*-test was used to examine differences in the mean scale score of attitude and preparedness between participants, according to gender (male/female), study level (fifth/sixth year), familiarity with the term deprescribing (yes/no), exposure to deprescribing in the required pharmacy curriculum (yes/no), and intended primary employment setting after graduation.

For analysis purposes, the intended primary employment setting after graduation was regrouped into direct patient care (including chain/independent community pharmacy, hospital and clinical pharmacy, eldercare/home care facility, and long-term care) and indirect patient care (including academia/research, government agency, pharmaceutical industry, and managed care/consultant/administration).

All scales demonstrated acceptable reliability using Cronbach’s Alpha test (attitude = 0.762, non-experiential curriculum preparedness = 0.847, self-perceived preparedness to deprescribe = 0.831). A two-sided *p*-value < 0.05 was considered statistically significant for all analyses.

### 2.5. Ethical Consideration

Ethical approval from the Research Ethics Committee at King Khalid University was obtained (KKU-193-2025-31). The research was conducted in accordance with the Declaration of Helsinki. All appropriate measures were taken to ensure the privacy and confidentiality of participants. The research objective, purpose, and data governance were clearly explained on the first page of the questionnaire. Participants indicated their agreement to participate in the study by proceeding to complete the survey.

## 3. Results

### 3.1. Background Characteristics of Study Participants

A total of 367 students from 23 universities/colleges participated in the study (Table 1). The sample showed a near-equal gender distribution between females (54.0%) and males (46.0%). Fifth-year students accounted for 62.1% of participants. Clinical pharmacy was the most frequently reported primary career plan after graduation (40.2%), followed by hospital pharmacy (20.8%) and academia/research (13.7%).

**Figure 1:**
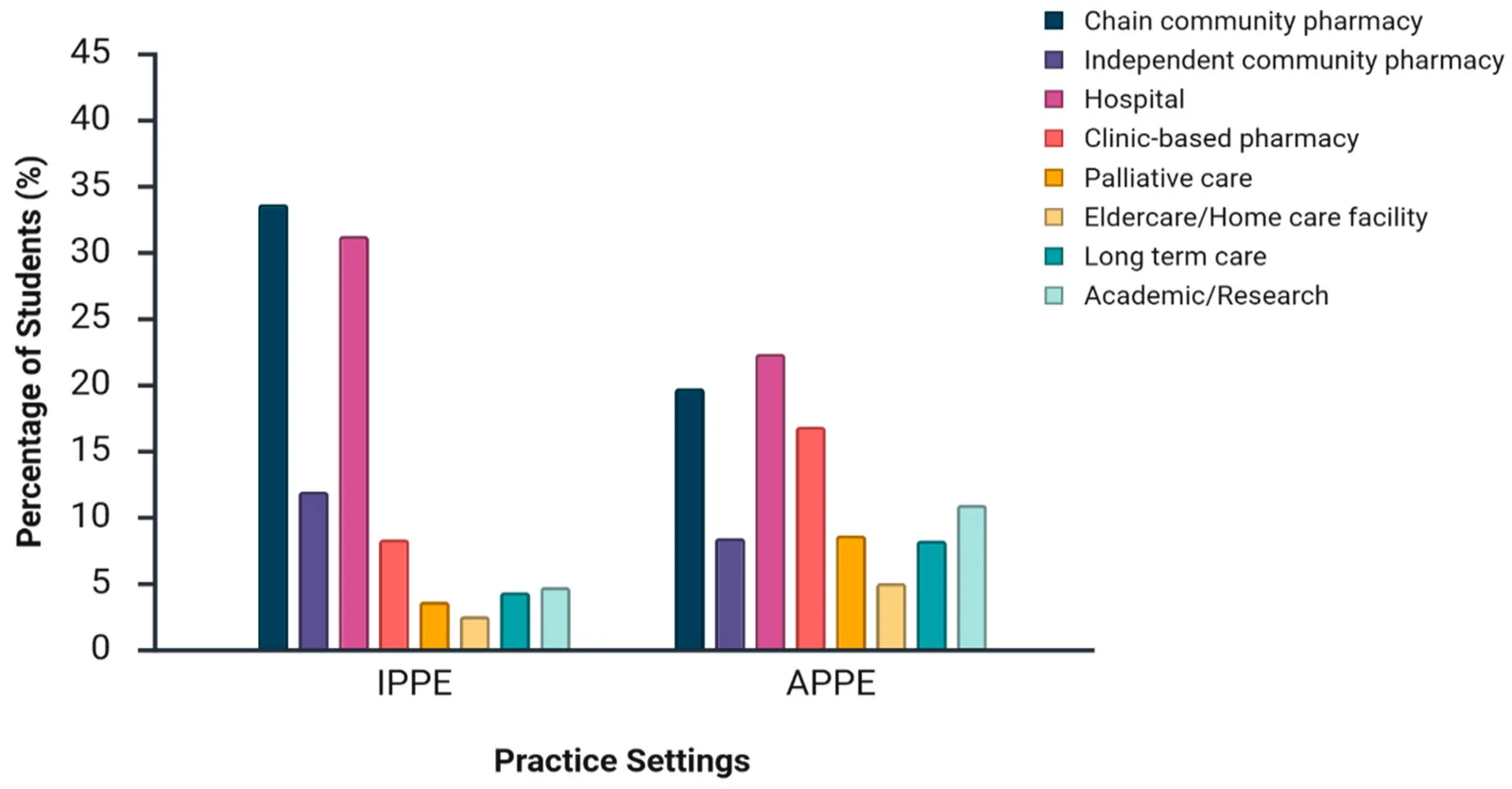
Pharmacy practice settings in which participants spent their IPPE (Introductory Pharmacy Practice Experience) and APPE (Advanced Pharmacy Practice Experience) (N = 367).

Figure 1 illustrates the practice settings in which participants spent their IPPE and APPE rotations. The most reported IPPE settings were chain community pharmacy (33.6%, *n* = 323), hospital pharmacy (31.2%, *n* = 300), and independent community pharmacy (11.9%, *n* = 114). Similarly, during APPE, the top three settings were hospital pharmacy (22.3%, *n* = 306), chain community pharmacy (19.7%, *n* = 270), and clinic-based pharmacy (16.8%, *n* = 230). Increased exposure, compared with IPPE, was observed in palliative care and long-term care.

### 3.2. Curricular Exposure to Deprescribing Principles

Familiarity with the term ‘deprescribing’ was reported by 55.3% of participants. Formal educational exposure was limited, with 36.2% reporting inclusion in the required curriculum and 21.3% in elective courses, while a significant proportion reported no exposure or uncertainty (Table 2).

### 3.3. Attitudes Towards Deprescribing

**Figure 2:**
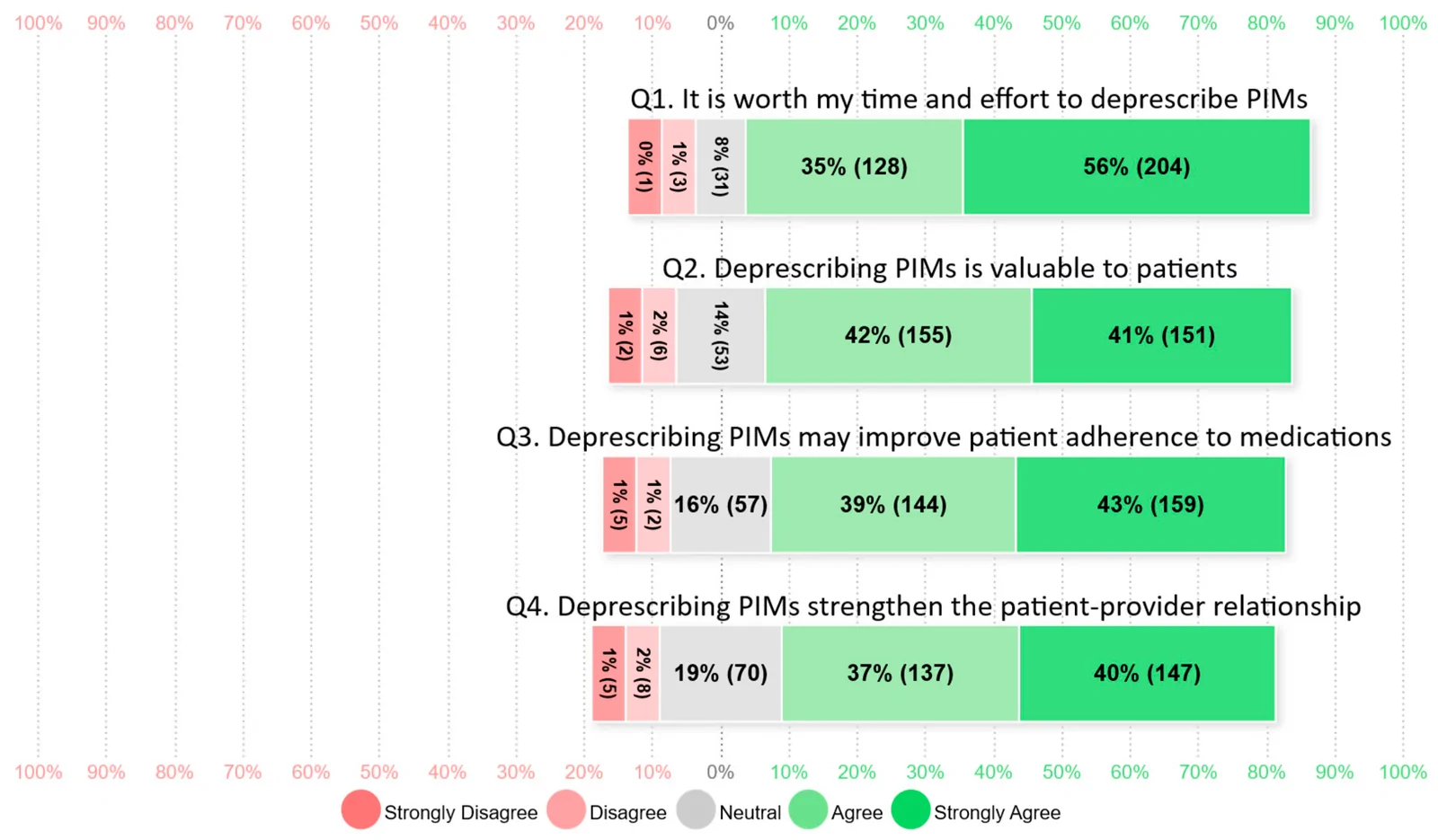
Pharmacy students’ attitude toward the benefits of deprescribing (PIMs = Potentially Inappropriate Medications) (N = 367).

Figure 2 demonstrates the participants’ attitude toward the benefits of deprescribing. Most participants strongly agreed or agreed that deprescribing PIMs is worth their time and effort (90.5%) and that it is valuable to patients (83.3%). Similarly, 82.5% strongly agreed or agreed that deprescribing may improve patient adherence to medications, whereas 77.4% strongly agreed or agreed it can strengthen the patient–provider relationship. The mean percentage score of agreement was 85.08%, demonstrating a highly positive attitude toward the benefits of deprescribing, while the mean scale score was 17.02 ± 2.65.

**Figure 3:**
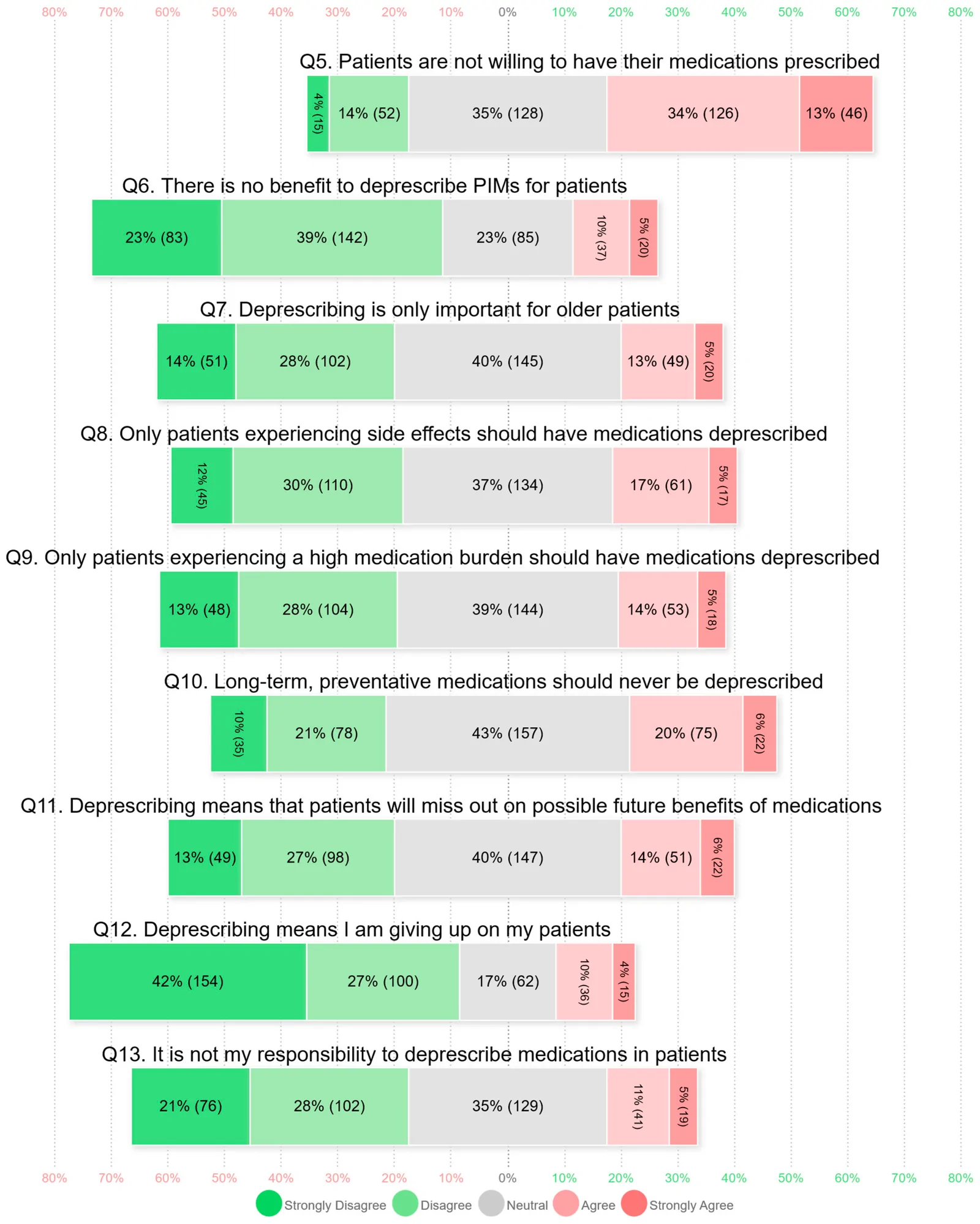
Pharmacy students’ attitude toward the misconceptions of deprescribing (N = 367).

Figure 3 demonstrates the participants’ attitude toward misconceptions about deprescribing. The highest levels of disagreement were reported for the misconceptions that deprescribing means giving up on patients (69.2%) and that there is no benefit to deprescribing PIMs (61.3%). Nearly half of the participants also strongly disagreed or disagreed that it is not their responsibility to deprescribe (48.5%), while 42.3% and 41.4% rejected the misconceptions that only patients experiencing side effects or a high medication burden, respectively, should have their medications deprescribed. Additionally, 41.7% strongly disagreed or disagreed that deprescribing is only important for older patients. The mean percentage score for this scale was 66.50%, demonstrating a moderately positive attitude toward misconceptions of deprescribing, while the mean scale score was 29.92 ± 6.71.

**Figure 4:**
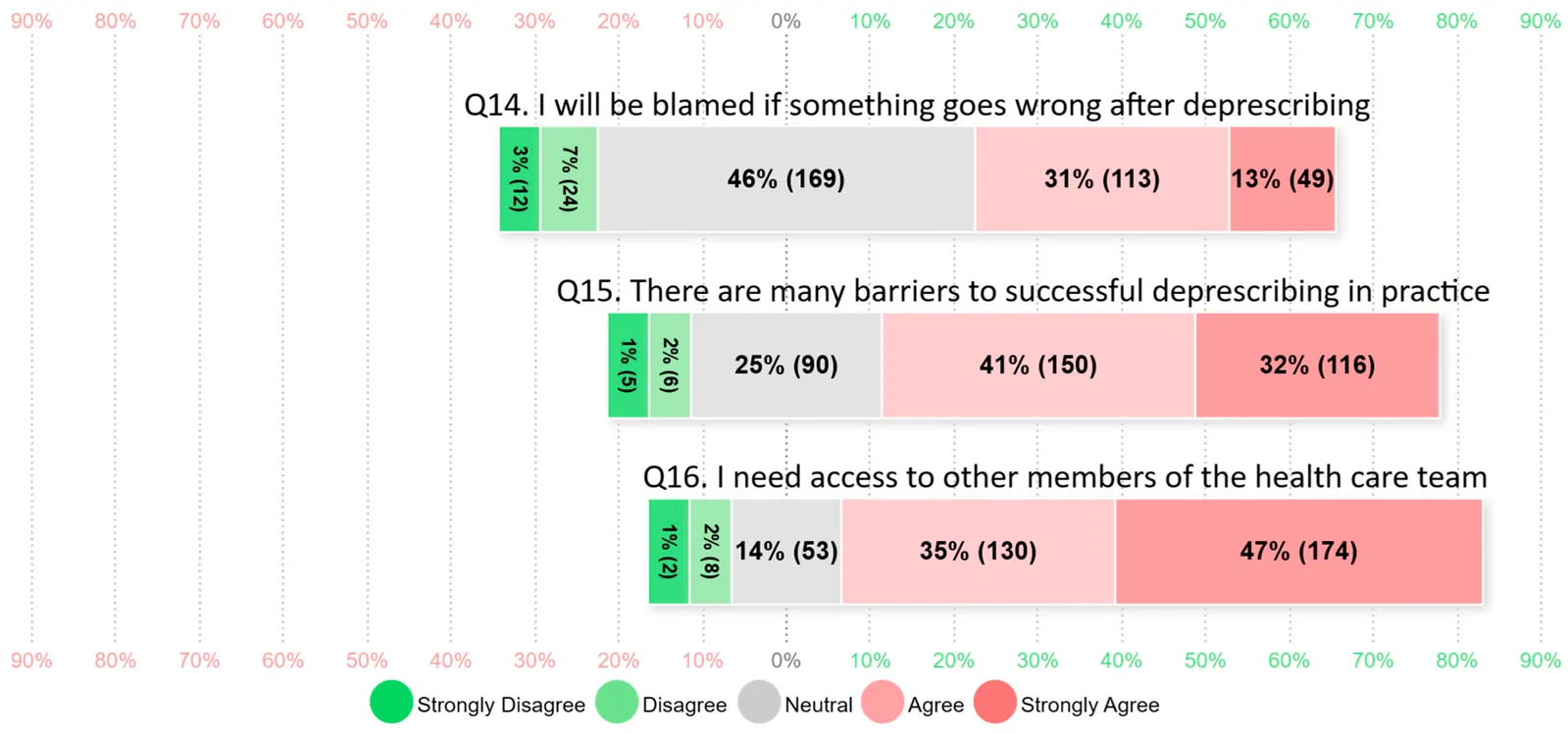
Pharmacy Students’ Attitude toward the Barriers of Deprescribing (N = 367).

Figure 4 demonstrates the participants’ attitude toward the barriers of deprescribing. Only 9.8% of participants strongly disagreed or disagreed that they would be blamed if something went wrong after deprescribing. Minimal disagreement was reported for the presence of barriers to successful deprescribing in practice (3.0%) and for the need to access other members of the healthcare team to support deprescribing (2.7%). The mean percentage score for this scale was 41.93%, indicating a low positive attitude toward the barriers of deprescribing, while the mean scale score was 6.29 ± 1.98.

### 3.4. Non-Experiential Curriculum Preparedness to Deprescribe

**Figure 5:**
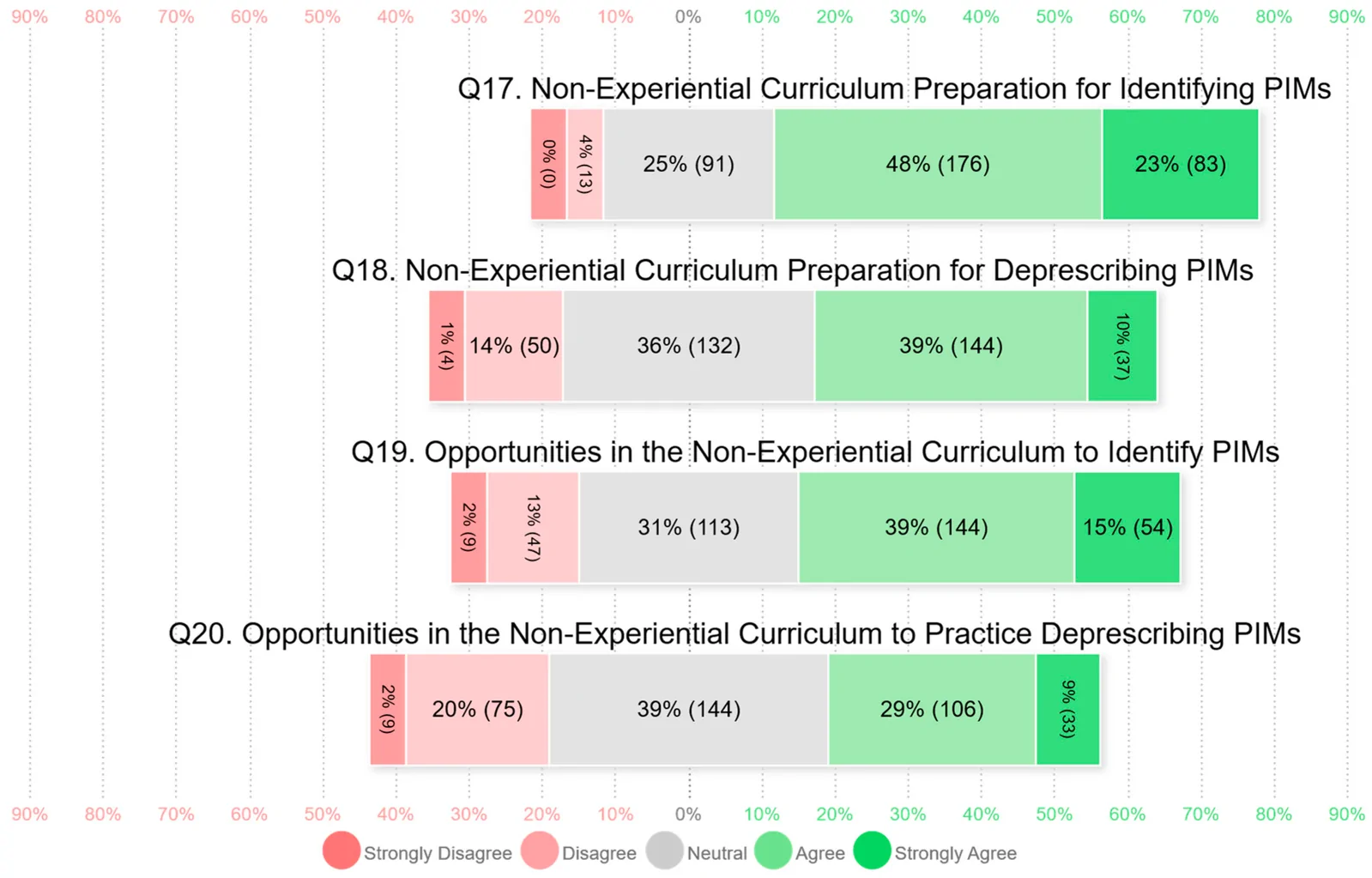
Pharmacy students’ perception of non-experiential curricular preparation and opportunities for identifying and deprescribing PIMs (N = 367).

Most participants strongly agreed or agreed that the non-experiential curriculum of their college prepared them to identify PIMs in practice (70.6%); however, only 49.3% strongly agreed or agreed that the non-experiential curriculum prepared them to deprescribe PIMs. In terms of practice opportunities, 53.9% reported agreement that they had opportunities to practice identifying PIMs, while a lower proportion (37.9%) strongly agreed or agreed that they had opportunities to practice deprescribing PIMs. The mean percentage score for this scale was 70.2%, indicating moderately positive perception toward non-experiential curriculum preparedness, while the mean scale score was 14.04 ± 3.03 (Figure 5).

### 3.5. Self-Perception of the Preparedness to Perform Deprescribing Steps

**Figure 6:**
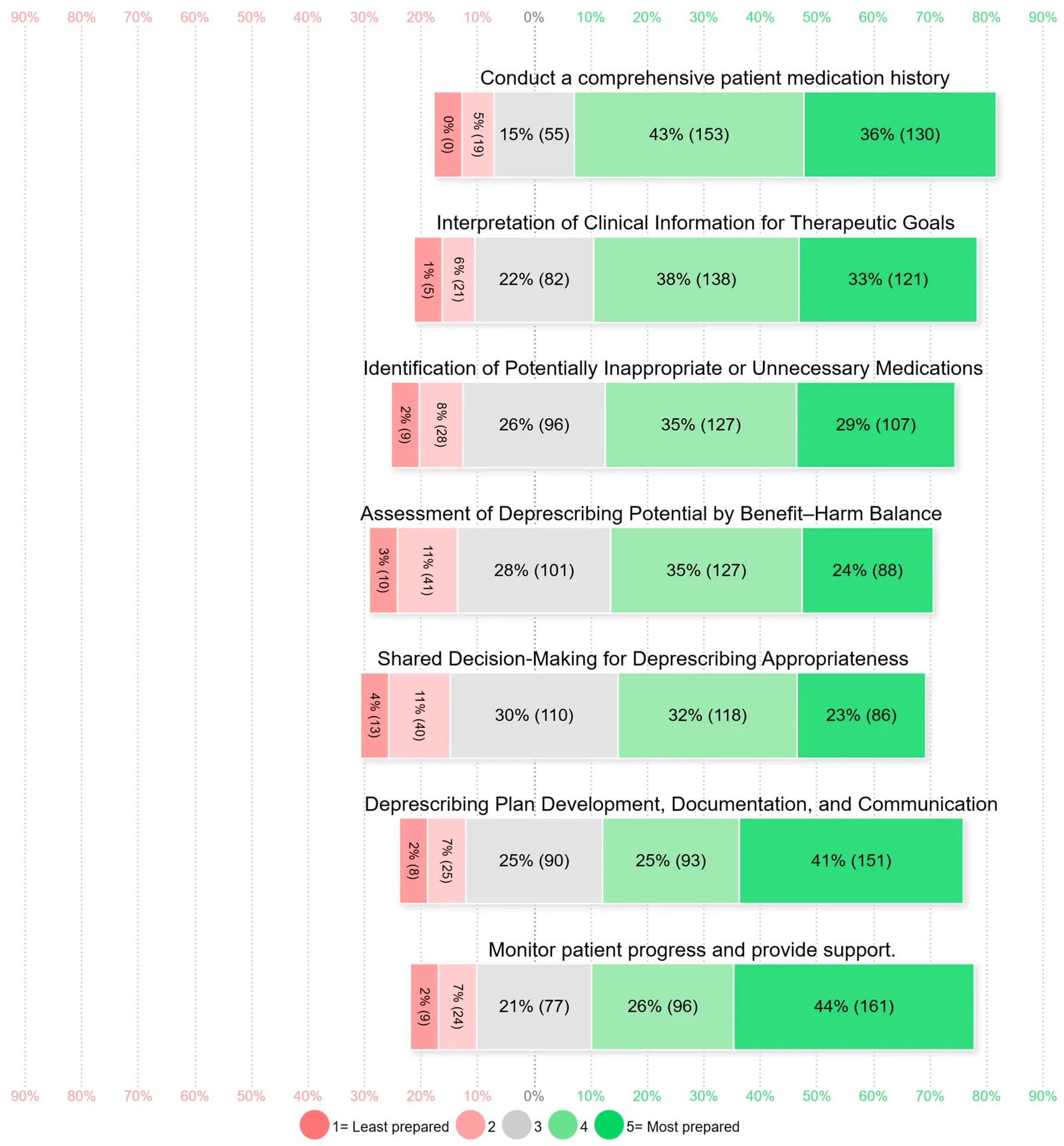
Pharmacy students’ self-perceived preparedness to implement the seven steps of the deprescribing process (1 = least prepared to 5 = most prepared) (N = 367).

Figure 6 demonstrates that the highest levels of perceived preparedness (ratings 4 and 5) were reported for conducting a comprehensive medication history (77.1%), interpreting clinical information in relation to therapeutic outcomes and goals (70.6%), and monitoring patient progress (70.1%). The lowest level of preparedness was reported for making shared decision-making judgments regarding deprescribing (55.6%). The mean percentage score for this scale was 77.24%, indicating moderate self-perception of the ability to perform deprescribing, while the mean scale score was 27.03 ± 5.07.

### 3.6. Interprofessional Roles in the Deprescribing Process

Table 3 shows that participants perceived clinical pharmacists as having the greatest role across most stages of the deprescribing process, including managing the deprescribing process (29.4%), undertaking medication review with older patients (26.7%), and identifying potentially unnecessary/inappropriate medications (25.8%). Physicians were most frequently identified as responsible for making the final decision to stop potentially unnecessary/inappropriate medications (33.3%), followed closely by clinical pharmacists (31.3%). Hospital pharmacists and community pharmacists were also recognized as contributors, particularly in identifying inappropriate medications, whereas nurses were less frequently selected across all stages.

### 3.7. Differences in the Attitude Toward Deprescribing, Non-Experiential Curriculum Preparedness and Self-Perceived Preparedness to Deprescribe Across Participant Characteristics

No statistically significant differences were observed in the mean score of attitude (benefits, misconceptions and barriers) among participants, however, statistically significant differences were observed in the mean score of perception of curriculum preparedness according to gender (*p* = 0.038), familiarity with the term deprescribing (*p* < 0.001), and exposure to deprescribing in the required pharmacy curriculum (*p* < 0.001) (Table 4). Additionally, no statistically significant differences in the mean score of self-perceived preparedness to deprescribe were found.

## 4. Discussion

This study identifies a notable gap in undergraduate pharmacy education in Saudi Arabia. Students showed a positive attitude toward deprescribing, reflecting recognition of its value in patient care. However, this positive attitude was not accompanied by adequate curricular exposure or strong perceived preparedness for the implementation of deprescribing in practice. Overall, the findings indicate that deprescribing is not yet established as a core clinical pharmacy competency, despite its direct relevance to medication optimization, patient safety, and personalized care.

The limited curricular exposure described in this study (36.2% and 21.3% in the required and elective curriculum, respectively) suggests that deprescribing is not yet fully incorporated into undergraduate pharmacy education in Saudi Arabia. In a UK study, Poots et al. also reported that medical and pharmacy students had limited practical experience with medication review and deprescribing [24]. Similarly, a study conducted in the US among medicine, pharmacy, and nursing trainees found gaps in curricular preparation for deprescribing and interprofessional role development [25]. Together, these findings support the need to move deprescribing education beyond theoretical exposure toward structured, practice-based training.

Despite this limited exposure, students in the present study showed highly positive attitudes toward the benefits of deprescribing. This is encouraging and aligns with previous research showing that pharmacy and other health professional students generally support deprescribing in principle [24,25]. However, recognizing deprescribing as valuable does not necessarily ensure readiness to implement it safely. Safe deprescribing requires structured training that develops confidence and applied skills in clinical reasoning, communication, shared decision-making, documentation, monitoring, and follow-up [26]. Therefore, deprescribing education should be incorporated as a competency-based component of pharmacy curricula and supported by practical teaching methods, including case-based learning, simulation, interprofessional activities, and performance-based assessment [26,27].

Students’ responses to the misconception items suggest that they understood some basic principles of deprescribing, but uncertainty remained around more complex issues, such as patients’ willingness to stop medications and the discontinuation of long-term preventive therapies. Similar concerns have been reported previously in the literature, where trainees may value deprescribing but feel uncertain when decisions involve benefit–harm assessment, patient preferences, and clinical ambiguity [14,24,25]. This indicates that teaching should move beyond definitions and expose students to realistic deprescribing scenarios involving multimorbidity, preventive medications, patient concerns, and changing therapeutic goals [26].

Another important finding was that students’ responses to the barrier-related items highlighted uncertainty about the practical implementation of deprescribing. Their concerns about blame, practical barriers, and the need to access other healthcare professionals suggest uncertainty about their role and responsibility in the deprescribing process. This is consistent with Saudi qualitative research conducted among physicians and pharmacists in secondary care hospitals, which explored healthcare professionals’ experiences of deprescribing older patients and identified barriers related to clinical responsibility, communication, organizational factors, and interprofessional collaboration [28]. Similar barriers have also been reported in cross-sectional studies, where limited confidence, unclear professional roles, and the need for interprofessional collaboration were identified as important factors affecting deprescribing practice [24,25]. Accordingly, pharmacy curricula should better prepare students to communicate deprescribing recommendations, document clinical decisions, manage uncertainty, and collaborate effectively with other healthcare professionals.

A key finding from this study was the gap between students’ ability to identify PIMs and their preparedness to implement deprescribing decisions. This gap is critical, as previous literature emphasizes that deprescribing requires more than recognizing an inappropriate medicine; it involves benefit–harm assessment, patient engagement, communication, documentation, and follow-up [26]. Consequently, deprescribing should be taught as a structured competency using practical and assessed learning methods rather than relying mainly on lecture-based teaching [23,29].

The interprofessional findings provide further insight into students’ understanding of deprescribing roles. Students recognized clinical pharmacists as key contributors to medication review, identifying inappropriate medications, and managing the deprescribing process, while physicians were viewed as central to final discontinuation decisions. However, the lower recognition of nurses and community pharmacists may suggest a narrower understanding of team-based roles in deprescribing. This is important because deprescribing is an interprofessional process that requires patient involvement, communication, monitoring, and collaboration across healthcare professions [26,30]. In addition, students exposed to deprescribing in the required curriculum had significantly higher perception of curriculum preparedness, although this did not translate into significantly higher self-perceived preparedness. This is supported by previous studies showing that students and trainees may have some awareness of deprescribing but still report limited practical experience, confidence, or preparedness to apply it in clinical practice [24,25]. This suggests that curricular exposure may improve awareness, but practical confidence requires more applied, experiential, interprofessional, and assessed learning activities.

This study has several strengths. It provides national-level insight into deprescribing education among senior pharmacy students in Saudi Arabia and includes a relatively large sample from 23 pharmacy colleges. It also examined multiple dimensions of deprescribing education, including awareness, curricular exposure, attitudes, misconceptions, perceived barriers, preparedness, and interprofessional roles. Together, these aspects provide a comprehensive understanding of students’ perceptions of deprescribing and identify key educational areas that should be addressed when integrating deprescribing into undergraduate pharmacy curricula.

However, some limitations should be acknowledged. The use of self-reported data may have introduced recall or social desirability bias, particularly in relation to students’ perceived preparedness and curricular exposure. Recruitment through Telegram groups may also have introduced selection bias, as students who were more interested in clinical pharmacy or medication safety may have been more likely to participate. Students’ perceived preparedness may not reflect their actual deprescribing skills. Therefore, future studies should assess students’ deprescribing competence using objective methods, such as clinical case scenarios, simulations, or observed practical assessments. Finally, this study provides a snapshot of the current status quo, serving as a valuable baseline for future research. Longitudinal or interventional studies are needed to examine whether and how students’ exposure, attitudes, and preparedness change over time.

## 5. Conclusions

This study shows that undergraduate pharmacy students in Saudi Arabia recognize the value of deprescribing, but their limited curricular exposure and moderate perceived preparedness indicate a need for stronger educational integration. Embedding deprescribing within undergraduate pharmacy curricula as a core clinical pharmacy competency, supported by structured, practical, and interprofessional training, is essential to prepare future pharmacists to contribute confidently and effectively to medication optimization and patient safety.

## Figures and Tables

**Table 1:** Demographic and academic characteristics of the study participants (N = 367).

Characteristic	Category	*n*	%
**Gender**	Male	169	46.0%
	Female	198	54.0%
**Age in years**	<22	48	13.0%
	22–24	290	79.1%
	≥25	29	7.9%
**Current Study Level**	Fifth year	228	62.1%
	Sixth year (Internship)	139	37.9%
**College of Enrollment**	Al Baha University	11	3.0%
	Alfaisal University	5	1.4%
	Buraydah Colleges	6	1.6%
	Imam Abdulrahman bin Faisal University	13	3.5%
	Jazan University	20	5.4%
	Jouf University	12	3.3%
	King Abdulaziz University	19	5.2%
	King Faisal University	11	3.0%
	King Khalid University	49	13.4%
	King Saud bin Abdulaziz University for Health Sciences	20	5.4%
	King Saud University	16	4.4%
	Najran University	13	3.5%
	Northern Borders University	4	1.1%
	Prince Sattam Bin Abdulazziz University	6	1.6%
	Princess Nourah bint Abdulrahman University	21	5.7%
	Qassim University	31	8.4%
	Shaqra University	5	1.4%
	Taibah University	9	2.5%
	Taif University	39	10.6%
	Umm Al Qura University	21	5.7%
	University of Hafr Al Batin	20	5.4%
	University of Hail	5	1.4%
	University of Tabuk	10	2.7%
**Current plans for primary employment after graduation**	Chain/Independent community pharmacy	23	6.3%
	Hospital pharmacy	76	20.8%
	Clinical pharmacy	147	40.2%
	Academia/research	50	13.7%
	Pharmaceutical Industry	40	10.9%
	Government agency	18	4.9%
	Managed care/consultant/administration	7	1.9%
	Other †	5	1.4%

† Other included Medical Representatives (*n* = 3), Pharmaceutical Company Business Roles (*n* = 2), and Unspecified (*n* = 1).

**Table 2:** Participants’ familiarity with deprescribing terminology and exposure to deprescribing instruction in required and elective pharmacy coursework (N = 367).

Variable	Yes		No		I Don’t Know	
	*n*	%	*n*	%	*n*	%
**Familiarity with the term ‘Deprescribing’**	203	55.3	164	44.7	0	0.0
**Deprescribing Instruction within the Required Pharmacy Curriculum**	133	36.2	168	45.8	66	18.0
**Deprescribing Instruction within Elective Pharmacy Coursework**	78	21.3	170	46.3	119	32.4
**If instruction on deprescribing was part of the curriculum at your college of** **pharmacy, during what curricular activities did this occur?**						
Lectures	171	49.6%	174	50.4%	-	-
Seminars (smaller groups than lectures and more interactive)	60	18.1%	271	81.9%	-	-
Use of simulators or other virtual environments	76	22.9%	256	77.1%	-	-
Problem based learning scenarios facilitated in groups	110	33.0%	223	67.0%	-	-
Student presentations	160	47.1%	180	52.9%	-	-
Informal teaching in clinical setting	113	33.5%	224	66.5%	-	-
Self-directed learning/reading/online materials	150	44.2%	189	55.8%	-	-
Gamification	44	13.4%	285	86.6%	-	-
Introductory Pharmacy Practice Experience	102	30.6%	231	69.4%	-	-
Advanced Pharmacy Practice Experience	90	27.2%	241	72.8%	-	-

**Table 3:** Perceived roles of professional groups in different stages of the deprescribing process (N = 367).

	Professional Groups									
	Physicians		Nurses		Clinical Pharmacists		Hospital Pharmacists		Community Pharmacists	
	*n*	*%*	*N*	*%*	*n*	*%*	*n*	*%*	*N*	*%*
**Undertake medication review with older patients**	303	23.0	173	13.1	352	26.7	266	20.2	222	16.9
**Identify potentially unnecessary/inappropriate medications**	251	18.5	151	11.2	350	25.8	315	23.3	287	21.2
**Make the final decision to stop potentially unnecessary/inappropriate medications**	315	33.3	49	5.2	296	31.3	158	16.7	129	13.6
**Manage the deprescribing process**	308	26.0	192	16.2	348	29.4	200	16.9	137	11.6

**Table 4:** Differences in the mean scores of attitude toward deprescribing, non-experiential curriculum preparedness, and self-perceived preparedness to deprescribe across participant characteristics.

Variables	Attitude Toward Deprescribing		Curriculum Preparation		Self-Perceived Preparedness	
	Mean ± SD	*p*-Value	Mean ± SD	*p*-Value	Mean ± SD	*p*-Value
Gender						
Male	53.61 ± 7.31	0.375	13.68 ± 3.04	0.038	27.47 ± 4.77	0.124
Female	52.90 ± 7.80		14.34 ± 2.99		26.66 ± 5.29	
Study year						
Fifth year	53.00 ± 7.46	0.468	14.07 ± 3.06	0.779	26.96 ± 4.76	0.752
Six year	53.60 ± 7.78		13.98 ± 2.98		27.14 ± 5.54	
Familiarity with the term deprescribing						
No	52.49 ± 7.50	0.095	13.26 ± 3.09	<0.001	26.88 ± 4.55	0.593
Yes	53.82 ± 7.60		14.66 ± 2.84		27.16 ± 5.46	
Exposure to deprescribing in the required curriculum						
No/I don’t know	52.82 ± 7.25	0.171	13.46 ± 2.89	<0.001	26.70 ± 4.60	0.113
Yes	53.95 ± 8.09		15.05 ± 3.02		27.62 ± 5.76	
Intended primary employment after graduation						
Direct patient care	53.40 ± 7.67	0.637	14.01 ± 3.04	0.872	27.01 ± 4.98	0.842
Indirect patient care	53.00 ± 7.36		14.07 ± 3.02		27.13 ± 5.26	

(SD): standard deviation.

## Data Availability

The original contributions presented in this study are included in the article/Supplementary Materials. Further inquiries can be directed to the corresponding author.

## Supplementary Materials

The following supporting information can be downloaded at: https://www.mdpi.com/article/10.3390/pharmacy14050124/s1, Study questionnaire.

## Abbreviations

The following abbreviations are used in this manuscript:

PIM	Potentially Inappropriate Medication
IPPE	Introductory Pharmacy Practice Experience
APPE	Advanced Pharmacy Practice Experience
