# Supplementary material for: A Cross-Sectional Analysis of Deprescribing Curricular Exposure, Attitudes, and Preparedness Among Undergraduate Pharmacy Students in Saudi Arabia
Source: Pharmacy (Basel). 2026 Aug 21;14(5):124. doi: 10.3390/pharmacy14050124 (PMC13516625; doi:10.3390/pharmacy14050124)
Supplement: Supplementary file 1 [file pharmacy-14-00124-s001.zip › pharmacy-4450838-supplementary.pdf]

The current state of deprescribing education in Saudi undergraduate pharmacy programs: a nationwide online survey of pharmacy students

We are a group of researchers in King Khalid University conducting a research project entitled "The current state of deprescribing education in Saudi undergraduate pharmacy programs: a nationwide online survey of pharmacy students".

The purpose of this project is to explore the curricular exposure and views of fifth- and sixth-year pharmacy students in the Saudi pharmacy programs about 'deprescribing'. We are inviting you because of you are a pharmacy student in their fifth- or sixth- year (internship) of study.

- Participation involves filling a survey that will take approximately 5-8 mins.
- Your participation in this research project is voluntary and you may choose not to participate or withdraw at any time.
- Your responses will be confidential, and we do not collect identifying information such as your name, email address or IP address.
- Responses will be anonymized and used only in aggregate form for research analysis.

By proceeding with this questionnaire, you confirm that:

- You have read and understood the purpose of the study.
- You consent to the use of your data for research purposes only, including publication of the findings of this study orally or in written form.

We highly value your time and effort in contributing to research that aims to improve pharmacy education and training.

If you have any questions or concerns regarding this study, please contact us at: leltayeb@kku.edu.sa

Thanks

* Indicates required question

Section one : Background information

1. At what college of pharmacy are you enrolled? *

Mark only one oval.

- King Khalid University
- Imam Abdulrahman bin Faisal University
- King Saud University
- Princess Nourah bint Abdulrahman University
- King Abdulaziz University
- King Faisal University
- King Saud bin Abdulaziz University for Health Sciences
- Qassim University
- Taibah University
- Najran University
- Al Baha University
- Jazan University
- University of Hail
- Riyadh Elm University
- Ibn Sina National College for Medical Studies
- Salman bin Abdulaziz University
- Alfaisal University
- Taif University
- Umm Al Qura University
- Prince Sattam Bin Abdulazziz University
- University of Tabuk
- Batterjee Medical College

- AlMaarefa University
- Fakeeh College for Medical Sciences
- Mohammed Al-Mana College for Medical Sciences
- Jouf University
- Northern Borders University
- Shaqra University
- Buraydah Colleges
- University of Hafr Al Batin
- Al Rayan National College of Health Sciences
- Other:

2. What is your Sex? *

Mark only one oval.

- Male
- Female

3. What is your age in years? *

Mark only one oval.

- 20
- 21
- 22
- 23
- 24
- 25 or more

4. At which study level are you currently enrolled? *

Mark only one oval.

- Level 9
- Level 10
- Level 11 and 12 (Internship)

5. What are your current plans for primary employment upon graduation from your college? *

Mark only one oval.

- Chain/Independent community pharmacy
- Hospital pharmacy
- Clinical pharmacy
- Academia/research
- Pharmaceutical Industry
- Government agency
- Managed care/consultant/administration
- Eldercare/Home care facility
- Long term care
- Other:

6. Please select from the following settings, where you have completed Introductory Pharmacy Practice Experience (IPPE) rotation? *

Note: IPPE often refers to the summer training

Mark only one oval per row.

	Yes	No
**Chain community pharmacy**	⬭	⬭
**Independent community pharmacy**	⬭	⬭
**Hospital**	⬭	⬭
**Clinic-based pharmacy**	⬭	⬭
**Palliative care**	⬭	⬭
**Eldercare/Home care facility**	⬭	⬭
**Long term care**	⬭	⬭
**Academia/research**	⬭	⬭

7. Please select from the following settings, where you have completed Advanced Pharmacy Practice Experience (APPE) rotations?

Note: APPE often refers to the internship

Mark only one oval per row.

	Yes	No
**Chain community pharmacy**	⬭	⬭
**Independent community pharmacy**	⬭	⬭
**Hospital**	⬭	⬭
**Clinic-based pharmacy**	⬭	⬭
**Palliative care**	⬭	⬭
**Eldercare/Home care facility**	⬭	⬭
**Long term care**	⬭	⬭
**Academia/research**	⬭	⬭

Section two: curricular exposure to deprescribing principles

8. Have you heard of the term 'deprescribing'? *

Mark only one oval.

⬭ Yes

⬭ No

From here onwards, we refer to Deprescribing as the process of planned and guided dose reduction, stopping or switching of medication that might be causing harm to the patient or no longer be of benefit.

9. Was there instruction (i.e., teaching) on deprescribing as part of your REQUIRED pharmacy curriculum? *

Mark only one oval.

- Yes
- No
- Don’t know

10. Was there instruction (i.e., teaching) on deprescribing as part of the ELECTIVE coursework at your college/school of pharmacy? *

Mark only one oval.

- Yes
- No
- Don’t know

11. If instruction on deprescribing was part of the curriculum at your college of pharmacy, during what curricular activities did this occur?

Mark only one oval per row.

	Yes	No
**Lectures**	◯	◯
**Seminars (smaller groups than lectures and more interactive)**	◯	◯
**Use of simulators or other virtual environments**	◯	◯
**Problem based learning scenarios facilitated in groups**	◯	◯
**Student presentations (e.g., SOAP)**	◯	◯
**Informal teaching in clinical setting**	◯	◯
**Self-directed learning/reading/online materials**	◯	◯
**Gamification**	◯	◯
**Introductory Pharmacy Practice Experience**	◯	◯
**Advanced Pharmacy Practice Experience**	◯	◯

Section three: perceptions of students about deprescribing

12. Please indicate your level of agreement with the following statements. *
(PIMs= Potentially Inappropriate Medications)

Mark only one oval per row.

	Strongly Agree	Agree	Neutral	Disagree	Strongly Disagree
**It is worth my time and effort to deprescribe PIMs**	⬭	⬭	⬭	⬭	⬭
**Patients are not willing to have their medications deprescribed**	⬭	⬭	⬭	⬭	⬭
**Deprescribing Potentially Inappropriate Medications (PIMs) is valuable to patients**	⬭	⬭	⬭	⬭	⬭
**Deprescribing PIMs may improve patient adherence to medications**	⬭	⬭	⬭	⬭	⬭
**Deprescribing PIMs strengthen the patient-provider relationship**	⬭	⬭	⬭	⬭	⬭
**There is no benefit to deprescribe PIMs for patients**	⬭	⬭	⬭	⬭	⬭
Deprescribing					

Deprescribing PIMs is safe					
Deprescribing is only important for older patients					
Only patients experiencing side effects should have medications deprescribed					
Only patients experiencing a high medication burden should have medications deprescribed					
Long-term, preventative medications should never be deprescribed					
Deprescribing means that patients will miss out on possible future benefits of medications					
Deprescribing means I am giving up on my patients					
I will be blamed if something goes wrong (return of [illegible]					

goes wrong (return of original condition, relapse or withdrawal symptoms) after deprescribing					
There are many barriers to successful deprescribing in practice	◯	◯	◯	◯	◯
It is not my responsibility to deprescribe medications in patients	◯	◯	◯	◯	◯
I need access to other members of the health care team other than my own discipline (e.g., physicians, pharmacists, nurses, etc.) to successfully deprescribe	◯	◯	◯	◯	◯

13. Please indicate your level of agreement with the following statements that are related to the non-experiential curriculum of your college. *

Note: non-experiential curriculum refers to classroom-based and simulated learning activities (such as lectures, labs, and case discussions) that occur within the pharmacy college and do not involve real-world practice placements or clinical rotations.

Mark only one oval per row.

	Strongly Agree	Agree	Neutral	Disagree	Strongly Disagree
**My college's non-experiential curriculum has prepared me to identify PIMs in practice**	⬭	⬭	⬭	⬭	⬭
**My college's non-experiential curriculum has prepared me to deprescribe PIMs in practice**	⬭	⬭	⬭	⬭	⬭
**My college's non-experiential curriculum has provided me the opportunity to practice identify PIMs**	⬭	⬭	⬭	⬭	⬭
**My college's non-experiential curriculum has provided** me the	⬭	⬭	⬭	⬭	⬭

me the opportunity to practice deprescribing PIMs

opportunity to practice deprescribing PIMs

Order the following steps of the deprescribing process from those you feel least prepared to address (1), to those you feel most prepared to address (5). Assume you had access to the patient, clinical assessments, and laboratory values.

14. Conduct a comprehensive patient medication history *

Mark only one oval.

	1	2	3	4	5	
Feel	◯	◯	◯	◯	◯	Feel most prepared to address

15. Interpret relevant information in the context of desired therapeutics outcomes and goals of care *

Mark only one oval.

	1	2	3	4	5	
Feel	◯	◯	◯	◯	◯	Feel most prepared to address

16. Identify medications that are no longer necessary, may have more harm than benefit, or are otherwise potentially inappropriate *

Mark only one oval.

	1	2	3	4	5	
Feel	◯	◯	◯	◯	◯	Feel most prepared to address

17. Assess the deprescribing potential of each medication by weighing benefits and harms *

Mark only one oval.

	1	2	3	4	5	
Feel	◯	◯	◯	◯	◯	Feel most prepared to address

18. Decide whether deprescribing a medication is appropriate using shared decision-making *

Mark only one oval.

	1	2	3	4	5	
Feel	◯	◯	◯	◯	◯	Feel most prepared to address

19. Design, document, and share a deprescribing and monitoring plan *

Mark only one oval.

	1	2	3	4	5	
Feel	◯	◯	◯	◯	◯	Feel most prepared to address

20. Monitor patient progress and provide support. *

Mark only one oval.

	1	2	3	4	5	
Feel	⬭	⬭	⬭	⬭	⬭	Feel most prepared to address

Interprofessional roles in the deprescribing process

21. In the deprescribing process, which professional groups undertake medication review with older patients? *

Mark only one oval per row.

	Yes	No
**Physicians**	⬭	⬭
**Nurses**	⬭	⬭
**Clinical pharmacists**	⬭	⬭
**Hospital pharmacists**	⬭	⬭
**Community pharmacists**	⬭	⬭

22. In the deprescribing process, which professional groups identify potentially unnecessary/inappropriate medications? *

Mark only one oval per row.

	Yes	No
**Physicians**	⬭	⬭
**Nurses**	⬭	⬭
**Clinical pharmacists**	⬭	⬭
**Hospital pharmacists**	⬭	⬭
**Community pharmacists**	⬭	⬭

23. In the deprescribing process, which professional groups make the final decision to stop potentially unnecessary/inappropriate medications? *

Mark only one oval per row.

	Yes	No
**Physicians**	⬭	⬭
**Nurses**	⬭	⬭
**Clinical pharmacists**	⬭	⬭
**Hospital pharmacists**	⬭	⬭
**Community pharmacists**	⬭	⬭

24. In the deprescribing process, which professional groups manage the deprescribing process? *

Mark only one oval per row.

	Yes	No
**Physicians**	⬭	⬭
**Nurses**	⬭	⬭
**Clinical pharmacists**	⬭	⬭
**Hospital pharmacists**	⬭	⬭
**Community pharmacists**	⬭	⬭

This content is neither created nor endorsed by Google.

Google Forms
